# Prevalence and predictors of alternative diagnoses on whole-leg ultrasound negative for acute deep venous thrombosis

**DOI:** 10.1186/s12880-020-00527-7

**Published:** 2020-12-02

**Authors:** Ebba Beller, Mattes Becher, Felix G. Meinel, Jens-Christian Kröger, Rengarajan Rajagopal, Raimund Höft, Marc-André Weber, Thomas Heller

**Affiliations:** 1 Institute of Diagnostic and Interventional Radiology, Paediatric Radiology and Neuroradiology, University Medical Centre Rostock, Ernst-Heydemann-Str. 6, 18057 Rostock, Germany; 2 Department of Radiodiagnosis, SMS Medical College, Jaipur, India; 3 Department of Emergency Medicine, University Medical Centre Rostock, Rostock, Germany

**Keywords:** Deep venous thrombosis, Duplex ultrasound, Compression ultrasound

## Abstract

**Background:**

To investigate the prevalence, spectrum, and predictors of alternative diagnoses explaining leg symptoms in patients negative for suspected acute deep venous thrombosis (DVT), which can be detected with whole-leg ultrasound.

**Methods:**

We retrospectively analyzed a cohort of 789 patients (median age 70 years, 50.6% women) evaluated with a whole-leg ultrasound examination for suspected acute DVT within one year. All findings in the radiology report were analyzed and electronic chart review was performed to collect clinical information.

**Results:**

Ultrasound was negative for acute DVT in 531 patients (67.3%). Among these, alternative diagnoses explaining leg symptoms were seen in 349 patients (65.7%). The most frequent alternative diagnoses were chronic venous insufficiency (147 patients, 27.7%), followed by lymphedema (48 patients, 9.0%) and chronic post-thrombotic changes (41 patients, 7.7%). Patients with alternative diagnoses were older (median 71 vs. 66 years, p = 0.0226), as well as more likely to present with leg swelling (39.5% vs. 23.1%, p = 0.0002), difference in leg circumference (25.5% vs. 14.8%, p = 0.0055) and redness (7.7% vs. 2.7%, p = 0.0213) than patients without alternative diagnosis. Independent predictors of finding alternative diagnoses on whole-leg ultrasound were older age (odds ratio 1.014 per year, p = 0.0119), leg swelling (OR 1.949, p = 0.0020) and history of previous DVT (OR 2.235, p = 0.0154).

**Conclusions:**

Alternative diagnoses explaining leg symptoms can be detected on whole-leg ultrasound in two thirds of patients with no evidence of acute DVT. Our data supports performing a comprehensive ultrasound evaluation beyond the venous system, particularly, in older patients, who present with leg swelling and a past history of DVT.

## Background

Acute deep venous thrombosis (DVT) is a common condition for which patients seek emergency medical care and is associated with pulmonary embolism as a potentially life-threatening complication. Therefore, accurate diagnosis and prompt initiation of anticoagulant therapy is important to reduce morbidity [1]. Clinical signs and symptoms of DVT are often vague [2] and laboratory D-dimer tests, routinely used for DVT screening, tend to have low specificity [3]. Duplex ultrasound of the lower extremity has therefore become the standard radiological screening test for patients with suspected acute DVT [4].

Some centers choose to perform a limited compression ultrasound examination as the initial test focusing primarily on the femoral and popliteal veins [5–8]. We prefer to perform a whole-leg ultrasound in all patients with suspected acute DVT in our institution, conforming to current multidisciplinary guidelines [4]. The major advantage of this approach, in addition to detection of isolated calf DVT, is that it allows to identify alternative diagnoses which may have caused the patient’s leg symptoms, thereby mimicking acute DVT [9–12].

In most cohort studies, the majority of focused ultrasound examinations for DVT in patients with suspected acute DVT are negative (56 to 77%) [13–15]. However, most of these patients had signs or symptoms that led to the clinical suspicion of acute DVT. For both health care providers and patients, it can be unsatisfactory to rule out DVT and discharge the patient without identifying a cause for the patient’s symptoms. Many alternative diagnoses such as chronic venous insufficiency (CVI) or soft tissue inflammation require specific treatment and it would be prudent to not miss these diagnoses during the initial work-up. Hence, evaluation for alternative diagnoses using whole-leg ultrasound in patients with negative acute DVT scans may enhance the overall quality of patient care as well as patients’ and health care providers’ satisfaction.

Therefore, we performed this study to investigate the prevalence, spectrum and predictors of alternative diagnosis explaining leg symptoms among patients evaluated with whole-leg ultrasound for suspected DVT.

## Methods

### Ethical approval, study design and patient selection

**Fig. 1:**
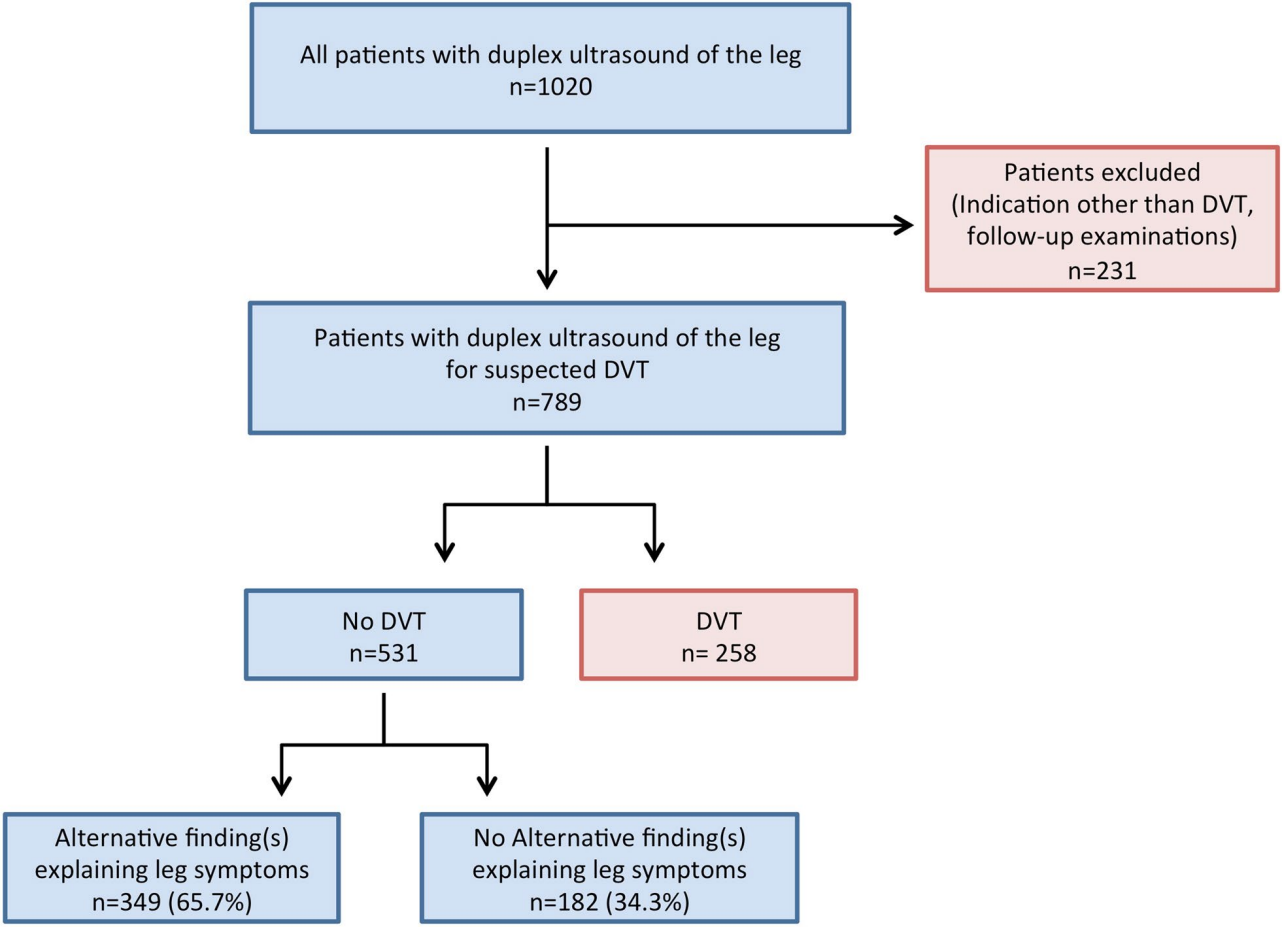
Flow chart of patient inclusion

The study was approved by our institutional review board with waiver of informed consent. The investigation was designed as a retrospective, single-centre cohort study. We included all patients (1) who were examined with whole-leg ultrasound (2) at our institution (3) between January 1 and December 31, 2014 (4) for suspected DVT (5) but no findings suggestive of acute DVT were seen on ultrasound. The year 2014 was chosen because during this year venous ultrasound evaluations were almost exclusively performed by two senior radiologists with extensive experience in vascular ultrasound. We excluded patients with (1) other indications for venous ultrasound and (2) follow-up examinations for established acute DVT diagnosed in the past 3 months (Fig. 1). All eligible patients were identified through a retrospective query of our radiology information system (Centricity 5.0, GE Healthcare).

### Ultrasound technique

We perform a whole-leg ultrasound of the symptomatic lower extremity in all patients with clinical suspicion of acute DVT in our institution as a combination of color-coded duplex ultrasound, compression ultrasound, and B-mode ultrasound from the groin to the lower leg by using a high-end ultrasound device (Aplio XG, Toshiba) and a linear transducer (PLT 604 AT, 6 MHz, Toshiba). In the case of suspicion of deep vein thrombosis of the iliac veins, the iliac veins are additionally examined using a convex transducer (PVT 375 BT, 3.5 MHz, Toshiba). The images are stored in the digital image archive (PACS, Agfa Impax 6.5.3). The ultrasound evaluation is performed or supervised by board-certified radiologists with subspecialisation in vascular and interventional radiology. Our protocol includes.

- compression ultrasound performed at 2 cm intervals from the common femoral vein to the ankle including the peroneal and posterior tibial veins in the calf,
- colour-coded duplex evaluation from the common femoral vein to the ankle,
- spectral doppler evaluation of the wave form in the common femoral vein (in case of abnormal waveform suggesting obstruction above the inguinal ligament, additional colour-coded duplex evaluation of the iliac veins is performed) and
- targeted ultrasound of symptomatic areas if symptoms are not explained by findings on standard thigh-to-ankle examination.

### Analysis of radiology reports

Radiology reports of the whole-leg ultrasound examination were retrospectively analyzed for the presence, type, location and presumed etiology of alternative diagnosis.

### Analysis of clinical data

Review of electronic patient charts was performed to record age, gender, presenting symptoms, risk factors, Wells scores and D-Dimer levels.

### Statistical analysis

Statistical analysis was performed with GraphPad Prism (version 8.4.2, GraphPad Software Inc). Continuous data were presented as median and interquartile range and compared using the nonparametric Mann–Whitney test. Categorical data were displayed as frequencies and proportions and compared between groups using Fisher’s exact test. We performed multiple logistic regression analysis to identify independent predictors of finding an alternative diagnosis in patients without acute DVT on ultrasound. Age and gender were entered into the model as predefined variables. Additionally, we included all variables with significant inter-group differences on univariate analysis. Due to collinearity between both variables, only leg swelling but not measured circumference difference was entered into the model. P values of < 0.05 were regarded as statistically significant.

## Results

### Patient characteristics

Our final study cohort consisted of 531 of patients with a whole-leg ultrasound negative for acute DVT, of which 269 (50.7%) were women. Patient characteristics are summarized in Table 1. Median age was 70 years (interquartile range 56–78 years). The most common local symptoms were leg pain (35.6%, 189 patients) and swelling (33.9%, 180 patients). 11.1% of patients had active malignancy and 11.9% of patients had a past medical history of DVT.

**Table 1:** Clinical and paraclinical predictors for alternative findings and no alternative findings in patients evaluated with whole-leg ultrasound for suspected DVT

	All patients without DVT (n = 531)		Alternative findings (n = 349)		No alternative findings (n = 182)		P value*
	n	%*	n	%*	n	%*	
Females	269	50.7	174	49.9	95	52.2	0.6478
Age in years, median (IQR^#^)	70 (56–78)		71 (58–78)		66 (52–77)		*0.0226*
Symptoms							
Leg pain	189	35.6	133	38.1	56	30.8	0.1047
Leg swelling	180	33.9	138	39.5	42	23.1	*0.0002*
Circumference difference	116	21.8	89	25.5	27	14.8	*0.0055*
Redness	32	6	27	7.7	5	2.7	*0.0213*
Risk factors							
Known coagulopathy	6	1.1	2	0.6	4	2.2	0.1878
Active cancer	59	11.1	39	11.2	20	11.0	0.9999
Previous DVT	63	11.9	50	14.3	13	7.1	*0.0160*
Wells score, median (interquartile range)	1 (0–2) [n = 168]		1 (0–2) [n = 117]		0 (0–2) [n = 51]		0.3508
Lab							
D-Dimer, median (interquartile range)	1.6 (0.73–3.1) [n = 319]		1.5 (0.78–2.925) [n = 204]		1.6 (0.68–3.35) [n = 115]		0.9119

*P values < 0.05 appear italic

^#^Interquartile range

### Prevalence and spectrum of alternative diagnosis

Alternative findings explaining leg symptoms were found on whole-leg ultrasound examination in 349 of 531 patients (65.7%, Fig. 1). The most common alternative findings were chronic venous insufficiency, found in 147 patients (27.7%), followed by lymphedema (48 patients, 9.0%) and post-thrombotic changes (41 patients, 7.7%). The complete spectrum of alternative findings is presented in Table 2.

**Table 2:** Prevalence and spectrum of alternative findings

Finding	Number of patients	% of all patients without DVT (n = 531)
Chronic venous insufficiency	147	27.7
Subcutaneous oedema, presumed lymphoedema	48	9.0
Post-thrombotic changes	41	7.7
Thrombophlebitis	37	7.0
Muscular injury/hematoma	34	6.4
(Ruptured) baker cyst	30	5.6
Peripheral artery disease	28	5.3
Subcutaneous oedema, aetiology not specified	20	3.8
Knee joint effusion/osteoarthritis/arthritis	17	3.2
Lymph node swelling	15	2.8
Soft tissue inflammation/erysipelas/cellulitis	15	2.8
Mass/tumour	7	1.3
Venous congestion/suspected heart failure	7	1.3
Ankle joint effusion/osteoarthritis/arthritis	5	0.9
Myositis	3	0.6
Arterial embolism	2	0.4
Bursitis	2	0.4
Others	5	0.9

### Comparison of patients with and without alternative diagnosis

Patients with alternative diagnoses were older than patients without alternative findings observed on whole-leg ultrasound (median 71 vs. 66 years, p = 0.0226) and more likely to present with leg swelling (39.5% vs. 23.1%, p = 0.0002), circumference difference (25.5% vs. 14.8%, p = 0.0055) and redness (7.7% vs. 2.7%, p = 0.0213). They were also more likely to have a previous history of DVT (14.3% vs. 7.1%, p = 0.0160). There were no differences in gender, proportion of cancer patients, D-Dimers and Wells score (Table 1).

### Multivariate analysis

On multiple logistic regression analysis (Table 3), age (Odds Ratio 1.014 for each one-year increment, p = 0.0119), leg swelling (OR 1.949, p = 0.0020) and previous DVT (OR 2.235, p = 0.0154) were independent predictors of finding alternative diagnoses in patients without acute DVT on whole-leg ultrasound.

**Table 3:** Multiple logistic regression analysis for the predictors of alternative findings vs. no alternative findings in patients evaluated with whole-leg ultrasound for suspected DVT

Predictor	Odds ratio	95% confidence Interval	P value
Age (per year)	1.014	1.003–1.025	*0.0119*
Male gender	1.118	0.7721–1.622	0.5547
Leg swelling	1.949	1.284–3.001	*0.0020*
Redness	2.089	0.8128–6.468	0.1555
Previous DVT	2.235	1.199–4.448	*0.0154*

P values < 0.05 appear italic

## Discussion

In this cohort study of patients negative for acute DVT on whole-leg ultrasound, alternative diagnoses were found in two thirds of the patients with CVI being the most common alternative diagnosis. Patients with alternative diagnoses were older, more likely to present with leg swelling, difference in leg circumference and redness than patients without an identifiable alternative diagnosis. Independent predictors of finding alternative diagnoses on whole-leg ultrasound were older age, leg swelling and a past history of DVT.

Few prior studies have investigated alternative diagnoses in patients with suspected acute DVT found on whole-leg ultrasound examination [9, 16, 17]. In these studies, alternative diagnoses were observed less often with whole-leg ultrasound, (11% to 31% of all patients with suspected DVT) [9, 16, 17] as compared to our study (44%). In all of these studies, ultrasound scans were either performed by ultrasound technicians [16]/sonographers [17], or by vascular technologists and interpreted by vascular surgeons [9]. This is in contrast to our study, here board-certified radiologists with subspecialisation in vascular and interventional radiology. performed or directly supervised the ultrasound examinations. It can therefore be speculated that the higher prevalence in our study may in part be due to radiologist-performed vs. non radiologist-performed ultrasound, since it is known that duplex ultrasound is highly operator dependent [18, 19]. The most common alternative diagnoses reported in these studies included chronic venous insufficiency [9], lymphadenopathy [16] and old thrombosis/post-thrombotic changes [17] similar to our study.

We assessed predictors for finding an alternative diagnosis on whole-leg ultrasound in patients suspected but negative for acute DVT. In a study to identify the risk factors for the common alternative diagnoses in patients with clinically suspected DVT (CVI, erysipelas, muscle rupture/hematoma and superficial venous thrombosis) [20] Cate Hoek et al. found that patients with CVI reported more often swelling of the entire leg having an insidious onset, as compared to patients with a confirmed diagnosis of acute DVT. Limb redness was seen as a distinctive feature in patients with erysipelas. Patients with muscle rupture/hematoma had neither swelling nor redness. Furthermore, patients with superficial venous thrombosis had tenderness on palpation of the offending vein [20]. However, all these four alternative diagnoses were based on clinical evaluation in a primary care setting and not based on whole-leg ultrasound findings as in our study [20].

In our analysis, older age, leg swelling and previous DVT were independent predictors of finding alternative diagnoses in patients without acute DVT on whole-leg ultrasound. A frequent complication of DVT includes the post-thrombotic syndrome (PTS) with a relatively high number of patients between 20 to 50% within 2 years of DVT diagnosis [21, 22]. Clinical manifestations of PTS typically include limb pain, heaviness, edema and pruritus [23]. PTS is a form of CVI that occurs due to chronic venous obstruction and damaged vein valves [21, 22]. Duplex ultrasound is the first imaging test of choice to evaluate for signs of CVI and post-thrombotic changes [24]. This might explain, why a past history of DVT was identified as risk factor for finding an alternative diagnosis on whole-leg ultrasound.

Since alternative diagnoses are relatively common in patients having a suspicion of acute DVT and these alternative diagnoses have important therapeutic consequences [10], focused sonographic evaluation of the venous system alone may not be sufficient in all patients with suspected acute DVT presenting with lower extremity symptoms. Useche et al. [10] state that in addition to whole-leg sonography in supine position, comparison of both extremities, performing specific maneuvers to elicit symptoms, as well as observing changes due to articular movement and switching to a standing position during the ultrasound examination might also be helpful. However, resources and time required may be limited in most emergency departments [16]. Nevertheless, the effort to establish an alternative diagnosis, when DVT is ruled out, seems particularly advisable in patients, who meet the following criteria: older age, leg swelling and previous history of DVT, which we identified to be independent predictors in our study. It should be kept in mind that patient compliance with follow-up ultrasound examinations after initial ultrasound negative for DVT seems to be extremely low [25], which also advocates the importance of performing a comprehensive initial ultrasound evaluation. As a practical implication of our study, we suggest to add targeted ultrasound of symptomatic areas if no DVT is found on standard thigh-to-ankle venous doppler ultrasound examination; particularly in older patients who present with leg swelling and a previous history of DVT.

This study has some limitations, which include its retrospective single-center nature. Symptomatic patients who presented to a university hospital were included in our analysis. This patient population may be different as compared to patients seen by primary care physicians. No external reference standard was available to confirm the findings. A more comprehensive prospective study is needed to determine the impact of alternative findings on subsequent diagnostic algorithm in patients and the benefits including costs in patient management.

## Conclusions

In summary, we found that alternative findings explaining leg symptoms can be detected on whole-leg ultrasound in two thirds of patients negative for acute DVT. Therefore, our study supports performing a detailed whole-leg ultrasound evaluation beyond the venous system (including targeted ultrasound of symptomatic areas) when negative for acute DVT. This may be particularly relevant in older patients, who present with leg swelling and who have a previous history of DVT, which were identified as independent predictors in our patient cohort.

## Data Availability

The datasets used and analyzed during this study are available from the corresponding author on reasonable request.

## Abbreviations

CVI: Chronic venous insufficiency
DVT: Deep venous thrombosis
OR: Odds ratio
